# Cervical cancer screening in women living with HIV attending primary care clinics in a health district, South Africa: a descriptive cross-sectional study

**DOI:** 10.11604/pamj.2022.43.32.33180

**Published:** 2022-09-20

**Authors:** Mmipe Lillian Saasa-Modise, John Mukuka Musonda, Joyce Sikwese-Musonda, Nomvula Josephine Maseko, Lineo Hlophe, Griffiths Kubeka

**Affiliations:** 1 Department of Family Medicine and Primary Health Care, University of Witwatersrand, Johannesburg, South Africa,; 2 Department of Family Medicine and Primary Health Care, University of Pretoria, Pretoria, South Africa,; 3 District Clinical Specialist Team, Ekurhuleni Health District, Germiston, South Africa,; 4 Aurum Institute, Johannesburg, South Africa

**Keywords:** Cervical cancer screening, uptake, human immunodeficiency virus, antiretroviral therapy, South Africa

## Abstract

**Introduction:**

globally, cervical cancer remains a public health problem. It is ranked the fourth most common cause of women's cancer. In South Africa, it was the second most common cancer diagnosed in 2012. The disease progresses rapidly in women living with Human Immunodeficiency Virus (HIV), due to immune suppression. The purpose was to evaluate cervical cancer screening in HIV-positive women attending primary health care (PHC) clinics in Ekurhuleni Health District (EHD), South Africa. Aim and Objectives: the study aim was to evaluate cervical cancer screening in HIV-positive women attending PHC clinics for routine care in Ekurhuleni Health District, Gauteng Province, South Africa. Objectives were to describe the cervical cancer screening uptake of HIV-positive women on antiretroviral therapy (ART) who remained in care and were screened, determine the length of time or period from starting ART to the first cervical cancer screening, and describe associations among screening, age, and the period from starting ART.

**Methods:**

this was a retrospective descriptive cross-sectional study design. A review of clinic records was conducted on women living with HIV and on antiretroviral therapy for at least four years. The study period was from March to September 2020. After a clustered randomization of clinics, 550 records were systematically selected. Stata version 16.1 was used for analysis.

**Results:**

the median age was 34, ranged 23-68, with the interquartile range (IQR) of 29-42 years. Nearly a third (32.9%, n= 181) had cervical cancer screening documented. Those with both an ART start date and first screening were 83% (n= 151). The median for ART duration was 5 years and ranged from 4-8 years. The median time to first screening was 43 weeks with an IQR of 16-67 weeks. Women aged 35-44 years or above 45 were more likely to be screened (AOR 3.2, CI: 1.0-9.8, p= 0.05) and (AOR 5.3, CI: 1.7-16.9, p= 0.01), respectively.

**Conclusion:**

there was suboptimal uptake and delay in initiating screening in women living with HIV. Nevertheless, the older women were, more likely to be screened. This study suggests poor adherence to policy and highlights the need for accelerated staff training on cervical cancer policy.

## Introduction

Cervical cancer is the fourth most common cause of cancer incidence and mortality in the world. Approximately 84% of incidents and 88% of deaths from cervical cancers occurred in lower-resource countries [1]. The South African (SA) estimated age-standardized incidence rate (ASIR) and age-standardized mortality rate (ASMR) were >40.0 and nearly 20.0 per 100 000 women, respectively. It is the second-highest cancer among women in South Africa. Early detection and prompt treatment of pre-cancerous lesions are the best protection against cervical cancer [2-4]. South Africa was the global epicenter of the HIV/AIDS epidemic with an estimated 7.97 million people living with HIV in 2019. Over one-fifth were women between 15 and 49 years old [5]. The country adopted the World Health Organization recommendation of screening women aged 30 to 55 years either through Papanicolaou tests (cervical cytology) every 10 years, or human papillomavirus (HPV) testing every 5 years with timely treatment of precancerous conditions. For women living with HIV, the South African policy on cervical cancer recommends screening be done at three-year intervals starting as soon as HIV is diagnosed [6]. South African data showed that women living with HIV had a higher prevalence of HPV infection than women who were HIV-negative because of sexual behavior and immune suppression due to HIV infection [7,8]. Evidence reveals that the incidence and progression of the squamous intraepithelial lesion (SIL) and cervical cancer could be reduced by early ART initiation and adherence [9-13]. Despite public awareness and health promotion activities to encourage women to screen, uptake remains low in South Africa. According to the District Health Barometer, 2017/2018 data, national cervical cancer screening coverage decreased from 64.5% in 2016/17 to 61.2% in 2017/18 against the national target of 70%. In the study district, cervical cancer screening coverage was lower than national, from 50.7% in 2018 to 45% in 2020 [14]. It was important to investigate whether cervical cancer screening was performed on all HIV-positive women who were receiving ART at primary health care clinics. Conducting research in this target population would reveal aspects of policy implementation which could improve clinical outcomes. The focus was on women living with HIV and on ART, which requires effective cervical cancer prevention strategies. Rationale: researchers observed poor performance and low uptake by the district from reports on cervical cancer screening. Besides, women living with HIV aged 30 years and below were not monitored nationally. Hospitals saw many women with advanced cervical cancer from primary health care clinics which suggested a lack of appropriate routine screening. Due to the high burden of HIV and AIDS in the country, we saw the relevance of investigating cervical cancer screening in women living with HIV. Conducting research on this target population would reveal possible gaps in cervical cancer screening in policy implementation.

## Methods

**Study design:** this was a descriptive cross-sectional and retrospective clinical records review of HIV-positive women.

**Setting:** the study was conducted in Ekurhuleni Health District, one of the five metropolitan municipalities in Gauteng Province, the economic hub of South Africa. The district was divided into three sub-districts, namely the North, East, and South. There were 93 primary healthcare facilities (86 small clinics, daily clinics and seven community health centres- CHCs) and six hospitals across the district. The North was serviced by 28 clinics, and one CHC, East by 26 clinics and three CHCs and South by 32 clinics and three CHCs and a population of 3,894,000 million who were predominantly black Africans [5].

**Study population:** clinical records of HIV-positive women presenting to primary health care clinics for routine HIV care during the study period.

**Selection of clinical records:** a sampled list of clinical records was generated through an electronic database called Tier.Net. The list was given to two administration clerks to retrieve records on the day of the data collection by four trained research assistants.

### Inclusion and exclusion criteria

**Inclusion criteria:** clinical records of HIV-positive women who were diagnosed with HIV at least four years previously and remained on care (ART). Also, 18 years and above.

**Exclusion criteria:** clinical records for HIV-negative women and/or those with an unknown HIV status. Incomplete information of less than 80% found in patient records.

**Sampling approach:** a review of clinic records of HIV-positive women, who were on antiretroviral therapy (ART) for at least the past four years and remained on care, was conducted from 01^st^ March to 30^th^ September 2020. Sampling was conducted stepwise. A clustered randomized sampling based on clinic size was done to include clinics which operated for 24-hours, 12-hours and 8-hours per sub-district. At each clinic, every tenth clinical record was randomly selected for the audit. This continued until the sample size was reached.

**Sample size:** the Raosoft sample size calculator was used to determine the number of files audited per clinic. A confidence level was set at 95%, a margin of error of 5% and a response distribution of 50%. The calculated sample size was 593.

**Data collection tool:** the questionnaire was developed in consultation with a statistician and public health experts. It was based on the South African cervical cancer screening guidelines. The information collected included demographic data, date of HIV diagnoses, initiation of ART, first screening for cervical cancer, repeat screening, never screened, CD4 count, and viral load.

**Pilot study:** involved 10 clinical records which were audited at Germiston Clinic to test and refine the tool for data collection. Data from the pilot study was not included in the final analysis of the study.

**Data collection process:** the research assistants visited participating clinics daily, obtained retrieved clinical records, and audited them according to the data collection tool. Where information was missing, the South African National Health Laboratory Service (NHLS) barcode was utilized to supplement it from their website. The information was captured by the researcher immediately. When data collection was completed, all clinical records were returned to the administration staff for filling and storage.

**Data analysis:** stata version 16.1 was used for data analysis. Details on the identification document, clinic name, participant age, HIV diagnosis, age group, date of HIV diagnosis, date when ART was commenced, date of first cervical cancer screening, and results were analyzed. A descriptive analysis using frequencies and percentages was done. The Chi-square test was used to evaluate the associations between categorical variables, ages, periods from the start of ART and cervical cancer screening. Univariate and multivariate logistic regression was used to test the strength of association between variables found to be statistically significant. The CD4 and viral load were also analyzed. Statistical significance was p-value < 0.05 and confidence interval 95%.

**Data storage:** once collected and analyzed, data was stored in a locked cupboard within the researcher´s office.

**Ethical considerations:** ethical approval was obtained from the Ekurhuleni District Research Ethics Committee, Gauteng Province. The approval for this study was No. 13/06/2019-06. The committee consisted of senior health managers and academics from the University of the Witwatersrand, University of Pretoria, Johannesburg University and Aurum Research Institute. Further permission was obtained from the health district Chief Director and facility managers of participating study sites. There were no human research respondents involved. Information collected was anonymous, confidential, and secured by the lead researcher.

## Results

**Clinical records an indication of cervical cancer screening performed in HIV-positive women:** the median age of women living with HIV and on ART was 34 years (IQR 29-42, ranging from 23 to 68 years). The median for ART duration among women from records surveyed was 5 years and ranged from four to eight (4-8) years. Out of a total of five hundred and ninety-three (593) records, forty-three had incomplete information. Only five hundred and fifty (550) clinical records were analyzed and nearly a third (32.9%, n=181) indicated that cervical cancer screening had been performed. Eighty-three percent (83%, n=151) had both ART start date and first cervical screening documented (Table 1).

**Table 1:** retrieved clinical records with indication of cervical cancer screening performed in women living with HIV

Sub-districts	Number of records retrieved	Number of records analysed	Records with cervical screening performed after HIV diagnosis
E1	72	64	32
E2	68	63	17
E3	56	54	17
N1	49	49	13
N2	97	72	31
N3	74	74	23
S1	62	61	8
S2	72	70	11
S3	43	43	29
Total	593	550	181

Comment: ninety-three percent of records retrieved were analysed; the rest of them had incomplete information Key: E=East; N=North; S=South

**Figure 1:**
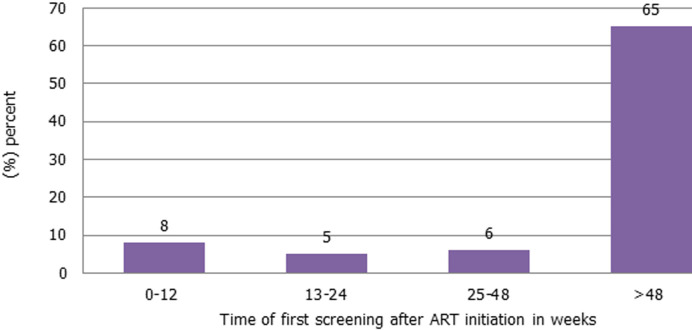
sources of data and the indication of cervical screening performed in women living with HIV

**Period from starting ART to first cervical cancer screening in women living with HIV:** sixty-five percent (65%) were screened after 48 weeks of ART initiation. Eighty percent (80%) were initiated early, at less than 12 weeks after diagnosis and treatment. Five (5) and six (6) percent were screened at 12-24 and 24-48 weeks, respectively (Figure 1).

**Figure 2:**
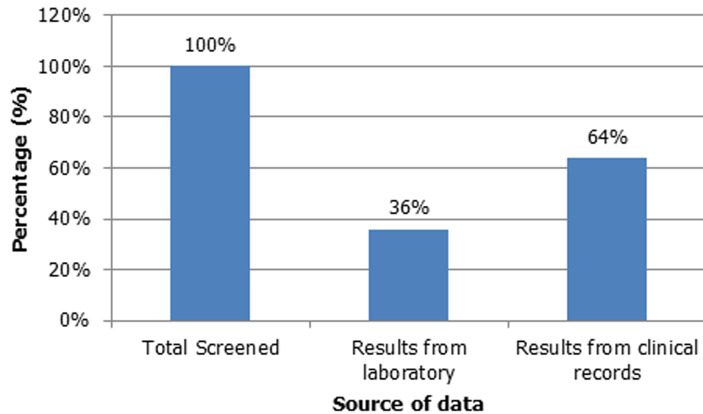
indication of the period from starting antiretroviral therapy to the first cervical cancer screening

**Associations of cervical cancer screening, age, and the period from starting ART to the first screening:** no association was observed between age cohorts 18 to 24, or 25 to 34 years, and cervical cancer screening. Women aged 35 to 44 and above 45 years (p-value 0.05, CI: 95%)and (p-value 0.01, CI: 95%), respectively, were significantly associated with cervical cancer screening. Women living with HIV were less likely to be screened after 2015. CD4 count was not associated with screening (Table 2). For those that were screened, there was a poor follow-up on cytology results. Not all results were obtained from clinical records (Figure 2).

**Table 2:** associations of cervical cancer screening, age, and the period from starting antiretroviral therapy to first screening

Variables		Unadjusted odds ratio (95% CI)	p-value	Adjusted odds ratio (95% CI)	p-value
Age group (years)	18-24	1.00	-	1.00	-
	25-4	2.7 (1.0-7.2)	0.04	2.4 (0.8-7.2)	0.12
	35-44	4.6 (1.7-12.5)	<0.001	3.2 (1.0-9.8)	0.05
	45+	6.0 (2.2-16.6)	<0.001	5.3 (1.7-16.9)	0.01
Period from starting antiretroviral therapy to first screening	Before 2015	1.00	-	1.00	-
	After 2015	0.5 (0.50-0.80)	0.001	0.62 (0.40-0.98)	0.04
CD4	≤250	1.00			
	201-350	0.90 (0.5-1.5)	0.68	-	-
	351-500	1.0 (06-1.8)	0.98	-	-
	501+	1.0 (0.6-1.8)	0.93	-	-

Women above 35 years old were more likely to have cervical cancer screening; after 2015, women living with HIV were less likely to have been screened

## Discussion

The study evaluated cervical cancer screening in women living with HIV on treatment and presenting for routine care at primary health care clinics in a health district in South Africa. Our findings showed nearly a third of women were screened for cervical cancer. Despite the national cervical cancer screening program having been introduced for many years, Ekurhuleni cervical cancer screening coverage was lower than the national target of 70%. It was 50.7% in 2018 and 45% in 2020 [15]. The suboptimal uptake of cervical cancer screening identified in this study is a concern, especially for women living with HIV and considered at high risk of developing cervical cancer. This study reports an uptake that is lower than 46.4% and 65.4% in research conducted in Limpopo and Soweto, respectively [8,9]. Surprisingly, more women living with HIV in Limpopo Province, rural South Africa, were screened than women in this study, yet the latter was an urban area and part of the economic hub of South Africa. The Limpopo findings may not be comparable to the current one because they used secondary data derived from all women regardless of their HIV status. The reasons for our findings could be attributed to nursing staff who did not offer cervical screening routinely because they were allocated other clinical duties and might have viewed it as additional work. Worse, demand for other primary care services was high and patient queues were long. The situation might discourage women who did not have a medical problem to wait for screening. Supervisors might not have focused on women living with HIV who were less than 30 years old because provincial indicators required reporting on those. Cervical cancer screening uptake for that age was expected because the South African cervical cancer policy recommended it though the country recently introduced a cervical cancer screening data collection tool for women living with HIV who are 20 years old and above. A descriptive study in Kenya to determine the uptake and factors to improve utilization of cervical cancer screening services reported a 23.1% uptake among women living with HIV. This was despite the high awareness of the development of cervical cancer [16]. Similarly, in Ethiopia, had an uptake of 18% and Cote d´Ivoire reported an uptake below two-thirds of women living with HIV at 59.7% [17,18]. The above variations could be based on national policies and geographical and cultural differences between those countries and South Africa.

The current study identified sixty-five percent (65%, n=118) of women had their first screening after 48 weeks of ART initiation. The finding is of concern and highlights non-adherence to the policy which recommends that HIV-positive women should be screened at diagnosis [6]. Challenges in this regard may be contextual and could have arisen from a lack of staff orientation on the relevant policy and understanding of practices. This was a missed opportunity because the nursing staff could not screen the most vulnerable women for cervical cancer. The long lapse from treatment to the first screening of almost a year (over 48 weeks) could compromise care and be undesirable. Our study focused on the period from starting treatment to first screening while the North Ethiopian study highlighted the length of time from diagnosis to screening [19]. Both studies showed the longer one remained in care the more likely to be screened. The finding reinforces the need to fast-track training, mentoring, and supervision of primary health care workers, particularly nursing staff, and primary care doctors. Also, it is important to reiterate that infrastructure for conducting cervical examination should be made available. Women living with HIV who were 35-44 years and those >45 years were four and six times more likely to have a cervical cancer screening test, respectively. A similar study in Cote d´Ivoire showed that age 45 years was strongly associated with the uptake of cervical cancer screening [19]. Additionally, a systematic review conducted in Ethiopia reported that the older age increased uptake of cervical screening by four-folds regardless of their HIV status [20].

In our study, the length of time had a significant association with cervical cancer screening (p-value 0.04, AOR 0.62, CI; 0.40-0.98). Women living with HIV on ART were less likely to take a cervical cancer screening test after 2015, as shown in Table 2. However, this may be read with caution. Among other reasons may be due to poor adherence to the cervical cancer screening policy by primary health care staff. In contrast to our findings, the Cote d´Ivoire study reported women were 1.4 times more likely to be screened. Women attending HIV clinics may have been advised by service providers to be screened because they were deemed vulnerable to developing cervical cancer [18]. The above findings may reflect changes in national ART initiation guidelines, logistics, or simply those chances of screening increased with the number of years one was on ART. The study found no evidence of cervical cancer screening based on baseline CD4 categories. Appropriately, the South African policy did not recommend screening based on CD4 count and ART.

**Study limitations and biases:** the study had some limitations. Being retrospective, the researchers could not draw any causal relationship between the uptake of cervical cancer screening and the reported associated factors. Women´s knowledge and understanding of cervical cancer screening may have increased the demand for screening. The study was done at primary healthcare clinics in a health district and results may not be generalized to the whole country because the circumstances may be different. We collected data from the past four years, which may suggest the possibility of missing other important information from the records prior to the study. Omission of undiagnosed or unknown HIV status of women and adolescents living with HIV who were excluded could have affected the study findings.

## Conclusion

There was suboptimal uptake of cervical cancer screening among women living with HIV and on ART. The PHC clinicians did not follow the prescripts of the policy to screen women living with HIV on diagnosis and every three years. The study revealed that women living with HIV and on ART of advanced age were more likely to be screened than younger women. Cervical cancer policy may have excluded younger women regardless of their HIV status. The study recommends qualitative research to be conducted to explore poor adherence to national cervical cancer screening policy. Moreover, it is imperative to advocate for accelerated staff training and monitoring of the cervical cancer policy implementation in all primary care clinics.

### What is known about this topic

- Women living with HIV are susceptible to cervical cancer;
- Uptake of cervical cancer screening is low in sub-Saharan Africa.

### What this study adds

- Reinforces the need to consistently offer cervical cancer screening services to all women who present to primary health care clinics;
- Cervical cancer screening services at primary health care clinic level may be hampered by sub-optimal record keeping, supervision and implementation of the national policy.
